# Rapid Screening of 20 Pesticide Residues in Tea by Thermal-Assisted Plasma Ionization–Time-of-Flight Mass Spectrometry

**DOI:** 10.3390/foods14193310

**Published:** 2025-09-24

**Authors:** Jiangsheng Mao, Weiqing Zhang, Chao Zhu, Wenjun Zhang, Mengmeng Yan, Hongxia Du, Hongwei Qin, Hui Li

**Affiliations:** 1 Institute of Quality Standard and Testing Technology for Agro-Products, Shandong Academy of Agricultural Sciences, Jinan 250100, China; maojiangsheng@163.com (J.M.); chaozhu@163.com (C.Z.); wenjunz@163.com (W.Z.); hongxiad@163.com (H.D.); hongweiq@163.com (H.Q.); 2 Guangzhou Hexin Instrument Co., Ltd., Guangzhou 510535, China; zhangweiqinghngy@126.com; 3 Guangdong MS Institute of Scientific Instrument Innovation, Guangzhou 510535, China; huili@163.com

**Keywords:** tea, pesticide residues, TAPI-TOF/MS, rapid screening, high-throughput

## Abstract

To achieve rapid screening and semi-quantitative analysis of pesticide residues in mobile laboratories and on-site tea testing, a novel method based on thermal-assisted plasma ionization–time-of-flight mass spectrometry (TAPI-TOF/MS) has been developed for the detection of 20 pesticide residues, including insecticides and fungicides, in tea. This method eliminates the need for liquid chromatography, or column connections. Instead, it utilizes the high temperature of the sample inlet and stage to fully volatilize and inject the sample. By integrating TAPI-TOF/MS with an automated pesticide residue pretreatment instrument, the entire sample extraction process can be performed automatically. The analysis time for each sample has been reduced to 1.5 min, allowing for the processing of 60 samples per batch. An accurate mass spectrometry database has been established for screening and confirmation purposes. The software automatically matches the mass spectrometry database by analyzing the measured ion mass deviation, ion abundance ratio, and the relative contribution weight of each ion, generating a qualitative score ranging from 0 to 100. The lowest concentration yielding a qualitative score of ≥75 was defined as the screening limit, which ranged from 0.10 to 5.00 mg/kg for the 20 pesticides. Within their respective linear ranges, the method demonstrated good linearity with correlation coefficients (R^2^) ranging from 0.983 to 0.999. The average recovery rates (*n* = 5) of the target pesticides ranged from 70.6% to 117.0% at the set standard concentrations, with relative standard deviations (RSD) ranging from 1.7% to 13.1%. Using this method, 15 tea samples purchased from the Rizhao market in China were analyzed. Ten samples were found to contain residues of metalaxyl or pyraclostrobin, yielding a detection rate of 66.7%. This technology provides technical support for the rapid detection and quality control of multiple pesticide residues in tea, meeting the requirements for high-throughput and on-site analysis.

## 1. Introduction

China is the world’s largest producer and consumer of tea, with tea ranking among the most widely consumed non-alcoholic beverages globally. With ongoing economic development and rising living standards, there has been a growing demand for high-quality tea products. This demand extends beyond the nutritional value of tea, placing increased emphasis on its safety for human consumption. Tea cultivation, however, faces significant challenges due to the susceptibility of tea plants to pests and diseases, which often leads to extensive pesticide use [1,2]. Improper and excessive application of pesticides can result in the accumulation of harmful residues, which may transfer to tea leaves, thereby compromising both product quality and consumer safety, and potentially posing risks to human health [3,4]. To address these concerns, numerous countries and international organizations have established maximum residue limits (MRLs) for pesticides in tea. For example, Japan’s Positive List System regulates 210 pesticide compounds related to tea [5], the European Union (EU) has set limits for 515 pesticide types [6], and China has established standards for 110 pesticide residues [7]. As concerns over food safety continue to rise, the scope of pesticide residue testing imposed by different countries is constantly expanding.

Currently, the primary techniques employed for detecting pesticide residues in tea include gas chromatography tandem mass spectrometry (GC-MS/MS) [8,9,10] and liquid chromatography tandem mass spectrometry (LC-MS/MS) [11,12]. These advanced instrumental methods are widely adopted by governmental regulatory authorities. To date, numerous studies have reported the screening and identification of pesticides in food using LC-TOF MS or LC-Orbitrap MS. In such studies, both GC and LC have been coupled with TOF MS [13,14,15,16,17,18,19,20]. These methods offer advantages such as accurate quantification, high sensitivity, and excellent stability. However, they often face challenges, including complex sample pre-treatment, lengthy analysis times, high detection costs, and the requirement for highly skilled operators, which restrict their applicability to laboratory-based quantitative analysis. Moreover, in actual tea production, most tea farmers manage small and scattered tea gardens (<0.067 hectares), and tea harvesting is typically concentrated within a short period, resulting in a brief market cycle and rapid product turnover. After harvesting, it is crucial for tea farmers to quickly determine whether the pesticide residue levels exceed the MRL. Tea that exceeds the MRL must be discarded, while tea that meets the MRL and is free from pesticide residues should be rapidly commercialized to ensure the delivery of fresh and safe products to consumers. Therefore, conventional instrumental analysis methods are not suitable for rapid on-site screening of pesticide residues in tea.

In recent years, the field of rapid detection of pesticide residues in food has experienced rapid development [21,22,23]. Currently, the primary rapid detection technologies available in the market include colloidal gold-based immunochromatographic assays [24,25,26], ambient ionization mass spectrometry [27,28], and enzyme-linked immunosorbent assays (ELISA) [29,30]. Colloidal gold-based immunochromatographic assays rely on the specific binding reaction between antigen and antibody. Due to their high efficiency, convenience, and low cost, these assays have been widely adopted in the safety monitoring of agricultural products. However, a major limitation is that each colloidal gold strip can only detect a single pesticide residue, which does not meet the growing demand for high-throughput detection in practical applications. Ambient ionization mass spectrometry enables nondestructive testing without requiring sample pretreatment, thereby significantly reducing analysis time. However, the presence of complex matrix components in samples may interfere with the ionization process, leading to inaccurate qualitative results and an increased rate of false positives. ELISA offers high specificity and can accurately identify specific pesticide molecules, minimizing interference from other substances. Although the procedure is relatively simple, it depends on antigen–antibody interactions, which may result in cross-reactivity and compromise detection accuracy. Furthermore, the results are highly sensitive to experimental conditions such as temperature, incubation time, and reaction duration. Therefore, strict control of experimental parameters is essential to ensure the reliability and reproducibility of the results. Previously, Wang, J.L. et al. [31] employed low-temperature plasma ambient ionization mass spectrometry (LTP-MS) and liquid chromatography coupled with triple quadrupole mass spectrometry (LC-TQ-MS), combined with organophosphorus magnetic molecularly imprinted polymers (OMMIPs)-based sample pretreatment, to achieve rapid screening of 90 pesticide residues across nine types of agricultural products. The main objective of their study was to develop an advanced sample pretreatment and purification methodology for pesticide residue analysis. The binding properties, magnetic separation capability, and reusability of the synthesized MMIPs were systematically evaluated. However, no comprehensive study has been conducted to assess the accuracy and precision of LTP-MS in high-throughput screening and detection of pesticide residues. Furthermore, the evaluation of the instrument’s detection performance has been limited to pesticide residues in crops with levels exceeding MRLs specified by the Chinese National Standard [7], with no investigation into the minimum detectable screening threshold. Additionally, the study focused exclusively on fruits and vegetables, where the matrix effects (ME) can vary significantly among different agricultural products. The matrix components present in tea are notably more complex than those found in fruits and vegetables [3,32]. Tea contains various natural constituents such as polyphenols, alkaloids, and pigments, which may co-elute with target pesticide analytes during instrumental analysis and compete for ionization in the mass spectrometry detection phase, thereby reducing ionization efficiency. For these reasons, the recently developed thermal-assisted plasma ionization–time-of-flight mass spectrometry (TAPI-TOF/MS) offers a comprehensive solution to the aforementioned challenges. Integrating TAPI-TOF/MS with a fully automated pesticide residue pretreatment system constitutes an advanced approach for the rapid screening and detection of pesticide residues, particularly suitable for mobile laboratories and on-site applications, thereby meeting the demands of high-throughput analysis.

However, there has been no reported application of this technology for the rapid screening of pesticide residues in tea [33,34]. This work conducts a comprehensive investigation from multiple perspectives, including the instrument’s working principle, data processing methodologies, and analytical performance. Based on the principles of atmospheric pressure ionization mass spectrometry, TAPI-TOF/MS integrates thermal desorption, plasma ionization, and TOF/MS technologies. It is specifically developed for the rapid screening of pesticide residues in food samples. Considering current pesticide application practices in tea cultivation and China’s MRLs for pesticides in tea, this study selected 20 commonly used pesticides as target analytes. These pesticides comprise certain highly toxic, prohibited substances, as well as several commonly utilized insecticides and fungicides. Detailed information regarding these pesticides is presented in Table 1. The application of TAPI-TOF/MS combined with automated pretreatment techniques for pesticide residue analysis was systematically investigated and optimized. A comprehensive mass spectral database of these 20 pesticide residues in tea was successfully established. Furthermore, rapid on-site screening of these 20 pesticide residues in tea samples was effectively achieved. In this study, the analysis time per sample was reduced to only 1.5 min, eliminating the need for complex sample pretreatment and chromatographic separation procedures. The method demonstrates high sensitivity, accurate qualitative identification, low detection costs, and the capability for semi-quantitative analysis. For pesticides with established MRLs, the software automatically compares the detected signals with the instrument’s spectral library and determines whether the MRL has been exceeded. The instrument is equipped with universal casters to enhance mobility. By installing and securing the system in a mobile laboratory vehicle, rapid on-site screening of pesticide residues in tea can be efficiently performed.

## 2. Materials and Methods

### 2.1. Reagents and Chemicals

Pesticide analytical standards were purchased from Dr. Ehrenstorfer (Augsburg, Germany), chromatographic grade acetonitrile, and methanol were obtained from Thermo Fisher (Waltham, MA, USA). Ultrapure water was obtained from Wahaha (Hangzhou, China). The QuEChERS analysis kit was purchased from Agilent Technologies (Agilent, Santa Clara, CA, USA), and it contains 150 mg of anhydrous magnesium sulfate (MgSO_4_), 50 mg of C_18_, 50 mg of primary–secondary amine (PSA), and 25 mg of graphitized carbon black (GCB). Green tea leaves were purchased from a market in Rizhao (China).

### 2.2. Instruments and Equipment

TAPI-TOF/MS (TAPI-TOF 1000, Guangzhou Hexin Instrument Co., Guangzhou, China), Automated Pre-treatment System for Pesticide Residues (Guangzhou Hexin Instrument Co., China), 6460 Triple Quad LC-MS/MS system (Agilent Technologies, CA, USA) equipped with a Poroshell 120 EC-C18 column (3.0 × 150 mm, 2.7 μm, Agilent Technologies, CA, USA), High-speed Refrigerated Centrifuge (Heal Force Bio-meditech Co., Shanghai, China), Micro-adjustable pipette (10–1000 μL, Eppendorf Group, Framingham, MA, USA), Electronic Balance (Precision: 1 in 10,000, Pioneer, OHAUS, Parsippany, NJ, USA), Vortex Mixer (SCI-VS, SCILOGEX, Rocky Hill, CT, USA).

### 2.3. Experimental Methods

#### 2.3.1. Preparation of Standard Solutions

Accurately weigh 0.010 g (accurate to 0.0001 g) of each of the 20 pesticide standards, dissolve them in acetonitrile, and dilute to a final volume of 100 mL to obtain a standard stock solution at a concentration of 100 μg/mL. Store this stock solution at −18 °C. Then, transfer 1 mL of the stock solution into a 10.0 mL volumetric flask and dilute it to the mark with acetonitrile to prepare a single standard working solution at a concentration of 10 μg/mL, which should be stored at 4 °C. Furthermore, the 10 μg/mL triazophos standard working solution was subjected to stepwise dilution with acetonitrile to yield a final concentration of 100.0 ng/mL, which was used as the instrument calibration solution.

#### 2.3.2. Extraction Method and Matrix Calibration Curves

This study employed the modified QuEChERS method, originally developed by Anastassiades et al. [35]. A blank tea sample, confirmed to be completely free of pesticide residues, was used. The tea sample and blank sample were ground into a fine powder. Subsequently, 2.0 g of the powdered tea and 2.0 g of NaCl were weighed into a 50 mL centrifuge tube. Then, 10 mL of distilled water was added, followed by vortex mixing for 1 min. The centrifuge tube was then placed in the test tube rack of the automated pre-treatment system for pesticide residues. The automated pretreatment system automatically adds 15 mL of acetonitrile as the extraction solvent to each centrifuge tube. The system performs an automated workflow that integrates vortex mixing, extraction, centrifugation, and pipetting. After selecting the appropriate program, the system initiated automatic processing. Upon completion of the processing, the instrument transfers 2 mL of the supernatant into a clean purification tube containing MgSO_4_, PSA, and GCB. The mixture is then vortexed for 1 min and centrifuged again at 4000 rpm for 5 min. Finally, 1.5 mL of the supernatant is transferred into a sample vial for TAPI-TOF/MS analysis.

After extraction, blank matrix extracts of tea leaves were collected and thoroughly mixed. The 20 pesticide standard solutions were then serially diluted using the mixed tea blank extract as the diluent, resulting in the preparation of matrix-matched standard solutions at concentrations of 0.10, 0.50, 1.00, 5.00, and 10.00 mg/kg. Matrix-matched calibration was employed for the quantification of all pesticide residues to effectively compensate for ME.

Following extraction, the prepared tea sample solution is automatically transferred into a 2 mL sample vial. The vial is then positioned in the designated sample area of the instrument. Upon opening the sample bottle cap, the instrument automatically assembles the pipette tips, aspirates the samples, and transfers them to the sample conveyor belt in a single, integrated operation. Under the combined effects of the high temperature of the heating stage and the vacuum siphon of the instrument, the samples rapidly volatilize, thereby completing the sample injection process. Subsequently, the system automatically discards the used pipette tips. Each sample has a volume of 10 μL and an analysis time of 1.5 min, allowing the system to process up to 60 samples in a single batch.

#### 2.3.3. Rapid Screening Mass Spectrometry and Output of Results

Upon activation of the mass spectrometer and prior to its use, the mass axis is calibrated using a 100.0 ng/mL triazophos calibration solution. A 1 mL aliquot of the standard solution is transferred into a sample vial, which is subsequently placed in the instrument’s tuning liquid area. The instrument automatically performs mass spectrum tuning, and sample analysis can commence only after the tuning process is successfully completed. The mass spectrometry analysis conditions are as follows: thermal-assisted plasma ionization source, positive ion mode, ion source temperature set at 90 °C, desolvation temperature maintained at 180 °C, helium flow rate adjusted to 1 L/min, cone voltage set at 120 V, collision voltage optimized to 46 V, and mass spectrometric scanning conducted in full scan mode across a mass-to-charge ratio (*m*/*z*) range of 72 to 1000.

Following sample collection, the instrument automatically performs mass spectrometry scanning to generate the mass spectrum. Subsequently, it automatically compares the acquired data with the pesticide mass spectrometry database and calculates the detection results. These results include the peak area of the quantitative ion, the concentration of pesticides in the sample, and the qualitative scores of all corresponding ions. If the qualitative scores of all ions for a specific pesticide exceed 75, the presence of that pesticide in the sample is confirmed. The system then automatically evaluates whether the detected pesticide residue exceeds the established MRL based on the measured concentration. When samples suspected of containing pesticide residues are detected, the traditional LC-MS/MS method, which is fully quantitative, is subsequently employed for verification to determine whether the pesticide residue levels exceed the regulatory limits.

#### 2.3.4. Operation Principle

A plasma ionization source functions as the ionization device. Under thermally assisted conditions, samples are converted into an aerosol state, subsequently ionized by the plasma ionization source, and then introduced into the time-of-flight mass spectrometer (TOF/MS) for qualitative and quantitative analysis. Specifically, helium is used as the working gas for the ion source. Helium, together with H_2_O and N_2_ from ambient air, is excited in a high-pressure alternating-frequency electric field to generate a high-energy plasma jet. During sample detection, analyte ions in the sample undergo thermal desorption to produce gaseous molecules or ions, which are ionized upon interaction with the low-temperature plasma (LTP) jet at the front end of the mass spectrometer before being introduced into the instrument for detection.

**Figure 1:**
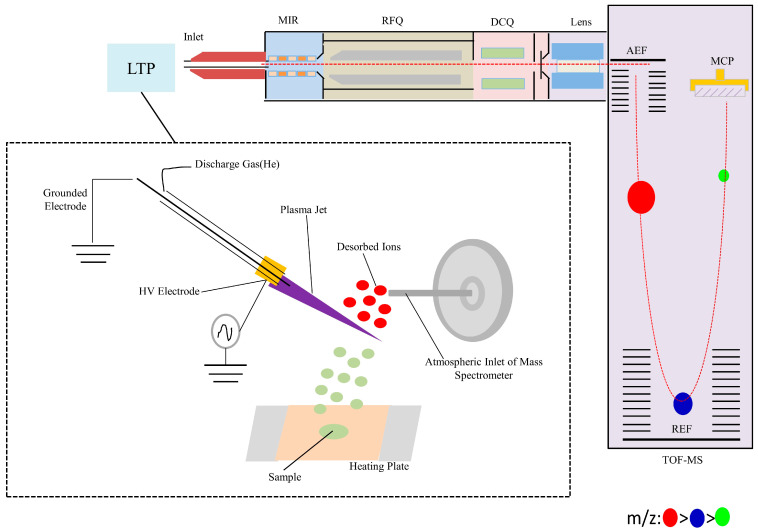
Schematic diagram of the ion source and instrument configuration. Low-temperature plasma (LTP), molecular ion reactor (MIR), radio frequency quadrupole (RFQ), direct current quadrupole (DCQ), acceleration electrode field (AEF), reflection electrode field (REF), microchannel plate (MCP).

The sample ions generated by the thermally assisted plasma ionization source are introduced into the molecular ion reactor (MIR) through a heated stainless steel capillary tube under the influence of an electric field. In a low-pressure environment, the ions are subjected to a strong radio frequency (RF) electric field and undergo numerous collisions with working gas molecules. These collisions result in a cooling and focusing effect that reduces both the kinetic energy dispersion and spatial divergence of the ions, thereby promoting molecular ion reactions and facilitating ion fragmentation analysis. The ions then enter the radio frequency quadrupole (RFQ), where they undergo further cooling and focusing through additional collisions. Subsequently, the ions are modulated by the combined electric fields of the direct current quadrupole (DCQ) and the ion optical lens before being directed into the modulation region of the mass analyzer. The mass analyzer adopts a vertical introduction structure, with the mass analysis and transmission regions arranged perpendicularly at a 90-degree angle. Upon being repelled by a pulsed voltage in the modulation region, the ions are accelerated through a double acceleration electrode field (AEF), reflected by a dual-reflection electrode field (REF), and finally detected by the microchannel plate (MCP) detector. A schematic diagram of the ion source and the instrument configuration is presented in Figure 1.

## 3. Results

### 3.1. Establishment of a Rapid Screening Mass Spectrometry Database and Analysis Results

**Figure 2:**
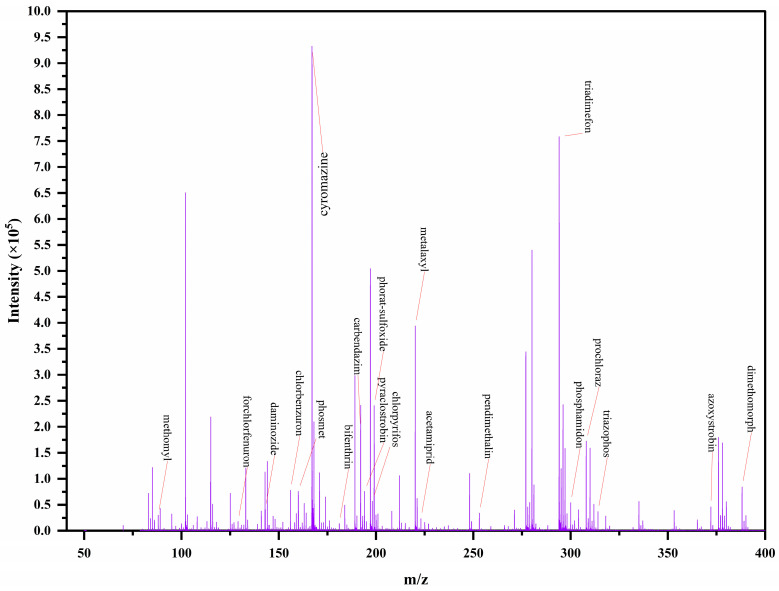
Mass spectrum of 20 pesticides. Tea blank matrix-matched pesticide standard solutions at concentrations of 5.00 mg/kg.

Following TOF/MS scanning of the samples, characteristic fragment ions corresponding to pesticides were selected as qualitative and quantitative ions based on literature and detected mass spectral data. The fragment ions with the highest abundance were designated as quantitative ions, while other characteristic ions were used as qualitative ions. Subsequently, a mass spectrometry database for 20 pesticides was constructed, incorporating ion abundance ratios, mass deviations, characteristic fragment ions, accurate *m*/*z* values, standard curve linear equations, and MRLs of each pesticide. The total ion chromatogram of the 20 pesticides is shown in Figure 2. After the scanning process, the samples undergo automated searching and precise matching against the established mass spectrometry database. This matching is based on the obtained ion mass deviation, ion abundance ratio, and the relative contribution weight of each ion. The system then automatically calculates a qualitative score ranging from 0 to 100. Due to the varying matrix compositions of different samples, the interfering impurities affecting pesticide determination vary accordingly. To mitigate matrix and solvent interference, all pesticides were quantified using matrix-matched calibration. To minimize the risk of false positive results, a matching score threshold of ≥75 was established as the criterion for suspected positive identification, based on reference to studies [36,37] and analysis of extensive experimental data, thereby enabling effective screening and identification of target compounds. When the qualitative score is below 75, the match between the detected pesticide components and the mass spectrometry database is considered insufficient to confirm the presence of the target compound in the sample. Therefore, any pesticide with a qualitative score of 75 or higher during the ion retrieval process is considered to be present in the sample.

In accordance with the MRLs for each pesticide, an appropriate concentration of the matrix standard solution was selected and calibrated using the external standard method. The quantitative ion peak areas, corresponding concentrations, and MRLs were then recorded in the mass spectrometry database. When scanning and analyzing unknown samples, the instrument software automatically calculates the quantitative ion peak area of the detected pesticides, compares these values with the data stored in the mass spectrometry database, and displays the pesticide content in the sample. It also automatically determines whether the pesticide residue levels exceed the established MRLs. Based on these determination results and in combination with the qualitative ion scores, the analyst is required to record only those pesticide residues with a qualitative ion score exceeding 75 and concentrations above the corresponding MRLs. The final determination of regulatory compliance is then made accordingly.

Pyraclostrobin is used as an example to illustrate the procedure for establishing a pesticide mass spectrometry database. Based on a comprehensive literature review [38], the following basic information was collected: CAS number: 175013-18-0, molecular weight: 387.82, chemical formula: C_19_H_18_ClN_3_O_4_, and SMILES notation: CON(C(=O)OC)C1=C(COC2=NN(C=C2)C3=CC=C(Cl)C=C3)C=CC=C1. The qualitative and quantitative ion pairs were determined to be 388.1/194.4 and 388.1/163.1, respectively. The qualitative ions were further confirmed as 194.0812 and 163.0628, along with additional qualitative data. In the first step, the aforementioned basic information is entered into the software, where the input of the SMILES string automatically generates the corresponding chemical structure. In the second step, qualitative data are entered by selecting the ion adduct form [M + 2H]^2+^, followed by the input of all relevant ions. For each ion, the weighting coefficients and abundance ratios are specified, ensuring that the total ion weights sum to 1. Among the ions, 194.0812, which exhibits high abundance, is designated as the quantitative ion, while 163.0628 serves as the qualitative ion. In the third step, quantitative data are entered by providing at least three distinct concentrations of the pyraclostrobin standard along with their corresponding peak areas. The software then automatically generates the calibration curve. The fourth step involves entering sample detection information. The sample category (fresh tea leaf), pesticide name (pyraclostrobin), and MRL value (10.0 mg/kg) are input into the software. The aforementioned steps are repeated to complete the data entry for 20 pesticides and their corresponding sample detection parameters.

### 3.2. Methodology Validation

The method was validated for linearity, ME, screening limits, accuracy, and precision in accordance with the European guideline document SANTE/11312/2021 (rev. 2, 2024) and Agricultural Industry Standard of the People’s Republic of China: guideline for the testing of pesticide residues in crops (NY/T 788-2018) [39]. Peak areas of ions were determined using an automated injection method, based on 20 types of tea matrix-matched pesticide standard solutions at varying concentrations The slope ratio between the matrix-matched and solvent-based calibration curves was calculated to assess the ME, using the formula: ME = [(slope in matrix/slope in solvent) − 1] × 100%. According to the test results, the ME values of the 20 pesticides in tea samples ranged from −86.2% to 78.8%, indicating that most pesticides were notably influenced by the tea matrix. Consequently, matrix-matched calibration curves were applied for all pesticides to mitigate the impact of matrix effects. Linear regression analysis was performed by plotting the mass concentration on the x-axis against the corresponding quantitative ion peak areas on the y-axis, resulting in linear equations and correlation coefficients (R^2^) for all 20 pesticides. Within their respective linear ranges, the 20 pesticides demonstrated excellent linear relationships, with correlation coefficients (R^2^) ranging from 0.983 to 0.999. The screening limits for these pesticides ranged from 0.10 to 5.00 mg/kg. The results of this method validation are summarized in Table 2.

Accuracy and precision were evaluated based on the average recovery of spiked samples. For the recovery rate evaluation, three concentration levels were established for each of the 20 pesticides based on their respective screening thresholds. Standard solutions of each pesticide were spiked into blank tea matrix samples to prepare matrix-matched calibration samples. For each compound, three concentration levels were tested in quintuplicate, yielding a total of 15 replicates per pesticide. Detailed results are presented in Table 3. The average recovery rates (*n* = 5) for the 20 pesticides ranged from 70.6% to 117.0%, with relative standard deviations (RSD) from five replicate measurements between 1.7% and 13.1%. Additionally, the qualitative scores for each pesticide exceeded 85 points, indicating reliable detection results for all tested pesticides.

According to the Agricultural Industry Standard of the People’s Republic of China: guideline for the testing of pesticide residues in crops (NY/T 788-2018) [39], the recovery rate for full quantitative analysis methods should fall within the range of 70% to 120%. Based on the test results, the recovery rates of all 20 pesticides met the specified requirements. However, during the experiment, it was observed that the recovery rates of certain pesticides were unsatisfactory, falling below 70% or exceeding 120%. Upon analysis, the primary cause was identified as the presence of various natural components in tea, such as caffeine, polyphenols, alkaloids, and pigments, which can induce a significant matrix effect. These substances may co-elute with the target pesticide analytes during instrumental analysis and compete for ionization during mass spectrometric detection, thereby reducing ionization efficiency and resulting in either ion suppression or enhancement, which can severely interfere with accurate quantification. Another contributing factor is the differing residue distribution behavior of systemic and contact pesticides in tea leaves, which stem from their distinct physicochemical properties [40,41,42]. Most systemic pesticides remain inside the tea leaf cells, making their extraction during sample pretreatment challenging. This difficulty in extracting pesticide residues results in relatively low recovery rates for certain pesticides in tea leaves. To ensure reliable screening results and avoid both false negatives due to undetected residues and false positives due to exaggerated response values, any sample containing detectable pesticide residues is classified as a suspected positive and must be confirmed using the full quantitative LC-MS/MS method.

Additionally, compared with traditional mass spectrometry analysis methods, this method uses a compact and portable mass spectrometer for analysis and detection. Although the miniaturization of the mass spectrometer and its vacuum system may slightly affect detection performance, the results demonstrate that the sensitivity for pesticide detection in the complex tea matrix is comparable to that of conventional benchtop instruments. Despite the fact that certain individual pesticides exhibit lower detection sensitivity and recovery rates than those achieved by traditional LC-MS/MS, this method remains suitable for semi-quantitative screening purposes and effectively meets the requirements for rapid on-site analysis.

In summary, the rapid screening and semi-quantitative method for detecting 20 pesticides in tea using TAPI-TOF/MS offers significant advantages over traditional methods in terms of analytical speed and reduced solvent consumption, while maintaining comparable detection sensitivity. This approach is well aligned with current trends in modern analytical chemistry that emphasize efficiency and environmental sustainability.

### 3.3. Sample Test

To verify the differences between semi-quantitative and full quantitative analytical methods, 15 different tea samples purchased from the market were analyzed using both approaches. The results are summarized in Table 4. These samples were labeled sequentially as Sample 1 to Sample 15 and were pretreated following the procedure outlined in Section 2.3.2. The resulting tea matrix extracts solution were then used as test solutions for instrumental analysis. Following analysis by TAPI-TOF/MS and LC-MS/MS, the peak areas of detected pesticide residues and their corresponding concentrations were meticulously recorded. Samples with no detectable pesticide residues were classified as negative, whereas those with detectable residues were classified as positive. Comparative analysis of the data demonstrates that the semi-quantitative and fully quantitative results fall within the same concentration range. This indicates that the semi-quantitative method employed in this study is both rapid and accurate, offering a reliable scientific basis for the rapid screening of pesticide residues in tea.

Based on the TAPI-TOF/MS and LC-MS/MS analyses, out of 15 tea samples tested, 10 were found to contain pesticide residues, while 5 were free from detectable residues. The detection rate of tea samples containing pesticide residues was 66.7%. Among the targeted analytes, 20 pesticides—including acetamiprid, azoxystrobin, and bifenthrin—were not detected in any of the samples. Metalaxyl was detected in two samples, with concentrations ranging from 0.144 to 0.176 mg/kg as determined by TAPI-TOF/MS and from 0.141 to 0.180 mg/kg by LC-MS/MS. Pyraclostrobin was detected in nine samples, with concentrations ranging from 0.197 to 0.855 mg/kg as determined by TAPI-TOF/MS and from 0.203 to 0.919 mg/kg by LC-MS/MS. The MRLs for pyraclostrobin in tea are set at 10 mg/kg in China, 0.1 mg/kg in the EU, and 25 mg/kg in Japan. For metalaxyl, MRLs have not been established for tea in China or Japan, whereas the EU has set an MRL of 0.05 mg/kg. According to these regulations, the concentrations of pyraclostrobin and metalaxyl in the 10 tea samples exceeded the MRLs established by the EU.

## 4. Discussion

The extraction of pesticide residues from samples is carried out using an automated pre-treatment system. This system is based on the QuEChERS extraction method, with modifications to the extraction procedure, and integrates all individual extraction steps into an automated pretreatment workflow, thereby enabling fully automated operation. The apparatus incorporates designated areas for centrifuge tube placement, sample vial positioning, solvent addition, centrifugation, and vortex mixing. It allows precise control over the volume of extraction solvent added, centrifugation duration, and vortex mixing time during the pre-treatment process. The traditional mass spectrometry analysis method typically involves connecting a mass spectrometer to both a high-performance liquid chromatography (HPLC) system and a chromatographic column. In this setup, the sample is introduced via the HPLC system and then separated within the chromatographic column. Compared to conventional LC-MS techniques, the proposed method reduces the analysis time from 60 min to 1.5 min and eliminates the need for HPLC coupling, or column separation steps. The use of the automated pre-treatment system significantly simplifies the sample preparation process, shortens detection time, and lowers labor costs.

The TAPI-TOF/MS is grounded in the principles of atmospheric pressure ionization mass spectrometry, integrating thermal desorption, plasma ionization, and time-of-flight mass spectrometry technologies. The sample extract solution is directly aspirated by a pipette tip and transferred to the sample conveyor belt in a single step. Subsequently, under the combined influence of the high temperature at the injection port and the heating platform, as well as the vacuum suction force generated by the instrument, the sample rapidly evaporated, thereby completing the thermal-assisted plasma ionization injection process. The combination of the TAPI-TOF/MS with the automated pesticide residue pre-treatment instrument not only achieves full automation of the sample extraction process, but also makes the process of injecting the samples into the mass spectrometer much faster. The analysis time for each sample has been reduced to 1.5 min, allowing for the processing of 60 samples per batch. The traditional LC-MS/MS method typically requires approximately 20 min to analyze a single sample [3,12]. In comparison, this method reduces the analysis time by a factor of 13, while significantly decreasing the consumption of toxic solvents and gases. Furthermore, the integration of a multifunctional fully automated pretreatment system and the development of the TAPI-TOF/MS rapid screening mass spectrometer enable on-site sample analysis. This eliminates the need for energy-intensive sample transportation and preservation processes, which often contribute to additional waste generation. Additionally, this method utilizes smaller sample volumes and requires fewer reagents, further minimizing waste production. Although the sensitivity of this method is slightly lower, it offers superior cost-effectiveness and environmental sustainability. Waste prevention and energy efficiency are key principles of green analytical chemistry [43,44]. Therefore, this approach aligns well with the principles of green analytical chemistry.

Following the scanning and analysis of the samples using TOF-MS, the instrument automatically searches a pre-established pesticide mass spectrometry database and accurately matches the analytical results with the corresponding entries. The database includes matrix-matched calibration curves, ion abundance ratios, ion mass deviations, the relative weight of each ion’s contribution, characteristic fragment ions, accurate *m*/*z* values, and MRLs for each pesticide. For each pesticide, the system automatically calculates a qualitative score (ranging from 0 to 100) and determines the corresponding residue level. A pesticide is considered present in the sample if its qualitative score is 75 or higher. If the residual amount exceeds the MRL, the system automatically classifies the product as non-compliant. By leveraging an established pesticide mass spectrometry database, this method enables both screening and confirmation without the need for reference standards. Furthermore, in accordance with the requirements of market supervision, the pesticide mass spectrometry database can be updated dynamically to incorporate new pesticide residues and categories of agricultural products. This continuous enhancement expands the database’s information content and broadens the scope of risk assessment for agricultural product quality and safety.

This semi-quantitative method exhibits high accuracy in both quantitative and qualitative analysis, as well as high sensitivity. To mitigate ME, matrix-matched calibration was employed for quantitative analysis. The screening limit for the 20 pesticides ranged from 0.10 to 5.00 mg/kg. Within their respective linear ranges, strong linear correlations were observed, with correlation coefficients (R^2^) ranging from 0.983 to 0.999. The average recovery rate (*n* = 5) of the target pesticides ranged from 70.6% to 117.0% at the set standard concentrations, and the RSD across five repeated measurements was between 1.7% and 13.1%. This innovative pesticide residue detection technology meets the requirements for high-throughput and rapid screening applications.

The distribution behavior of systemic and contact pesticides in tea, along with their physicochemical properties, are key factors influencing the efficiency of pesticide residue extraction in agricultural products. Physicochemical properties such as solubility, octanol-water partition coefficient, and vapor pressure determine how pesticides adsorb within tea matrices and how they behave during sample pretreatment. Systemic pesticide residues may be distributed within tea tissue cells, whereas contact pesticide residues are predominantly found on the surface of tea leaves. These differences contribute to variations in recovery rates. Generally, systemic fungicides exhibit relatively low recovery rates, including compounds such as azoxystrobin, dimethomorph, prochloraz, and triadimefon, while contact insecticides tend to show higher recovery rates, such as acetamiprid and triazophos. However, certain organophosphorus and pyrethroid insecticides, due to their low solubility, non-polar nature, and permeability, also exhibit poor recovery, including bifenthrin, chlorpyrifos, and phorate-sulfoxide. These observations provide insight into the relationship between pesticide characteristics and their extraction efficiency. Water-soluble, non-systemic, and contact-acting pesticides are more readily extracted from plant tissues. In contrast, systemic and penetrative (locally systemic) pesticides tend to persist within plant tissues for extended periods and are more difficult to extract effectively.

Fifteen tea samples obtained from the market were analyzed using TAPI-TOF/MS detection technology. The results demonstrated that this technology meets the requirements for rapid screening and semi-quantitative analysis of pesticide residues in tea. Although the market samples collected are limited, thereby restricting the generalizability of the findings, the study effectively addresses the analytical challenges associated with pesticide residue detection in tea, which involves complex matrices and demands rapid testing.

## 5. Conclusions

To the best of our knowledge, no previous studies have reported the application of TAPI-TOF/MS technology for rapid screening and semi-quantitative detection of pesticide residues in tea. The combined application of the automated pre-treatment system and TAPI-TOF/MS significantly reduces the sample processing and analysis time, lowers the false positive rate, and enables trace-level detection and simultaneous identification of multiple compounds. Equipped with a computer pre-installed with user-friendly data processing software, the instrument ensures efficient operation and meets the demands of high-throughput, rapid screening in mobile laboratories and on-site settings. In future work, this method should be applied to a larger number of tea samples to accumulate more comprehensive data, further enhance its performance, and expand the scope of detectable pesticides. In conclusion, this study presents an efficient and reliable approach for the simultaneous detection of multiple pesticide residues in agricultural products, offering new methodologies and technical support for agricultural product quality control, as well as novel insights into the advancement of mass spectrometry technologies.

## Figures and Tables

**Table 1:** Information on 20 types of pesticides.

Pesticides	Chemical Formula	Type	Family	CAS Number
Acetamiprid	C_10_H_11_ClN_4_	Insecticide	pyridylmethylamine	135410-20-7
Azoxystrobin	C_22_H_17_N_3_O_5_	Fungicide	strobilurin	131860-33-8
Bifenthrin	C_23_H_22_ClF_3_O_2_	Acaricide, insecticide	pyrethroid	82657-04-3
Carbendazim	C_9_H_9_N_3_O_2_	Fungicide	benzimidazole	10605-21-7
Chlorbenzuron	C_14_H_10_Cl_2_N_2_O_2_	Insecticide	chitin synthesis inhibitor	57160-47-1
Chlorpyrifos	C_9_H_11_Cl_3_NO_3_PS	Nematocide, acaricide, insecticide	organophosphorus	2921-88-2
Cyromazine	C_6_H_10_N_6_	Insecticide, acaricide	chitin synthesis inhibitor	66215-27-8
Daminozide	C_6_H_12_N_2_O_3_	plant growth regulator	plant growth regulator	1596-84-5
Dimethomorph	C_21_H_22_ClNO_4_	Fungicide	morpholine	110488-70-5
Forchlorfenuron	C_12_H_10_ClN_3_O	Plant growth regulator	plant growth regulator	68157-60-8
Metalaxyl	C_15_H_21_NO_4_	Fungicide	anilide	57837-19-1
Methomyl	C_5_H_10_N_2_O_2_S	Insecticide	carbamate	16752-77-5
Pendimethalin	C_13_H_19_N_3_O_4_	Herbicide	dinitroaniline	40487-42-1
Phorat-sulfoxide	C_7_H_17_O_3_PS_3_	Insecticide, miticide	organophosphorus	2588-03-6
Phosmet	C_11_H_12_NO_4_PS_2_	Insecticide	organothiophosphate	732-11-6
Phosphamidon	C_10_H_19_ClNO_5_P	Insecticide, miticide	organophosphorus	13171-21-6
Prochloraz	C_15_H_16_Cl_3_N_3_O_2_	Fungicide	imidazole	67747-09-5
Pyraclostrobin	C_19_H_18_ClN_3_O_4_	Fungicide	carbamate	175013-18-0
Triadimefon	C_14_H_16_ClN_3_O_2_	Fungicide	conazole	43121-43-3
Triazophos	C_12_H_16_N_3_O_3_PS	Nematocide, acaricide, insecticide	organophosphorus	24017-47-8

**Table 2:** Results of method validation.

Pesticide	Linear Range/ (mg/kg)	Matrix	Linear Equation	Correlation Coefficient (R^2^)	ME (%)	Screening Limit/ (mg/kg)	MRLs for China/ (mg/kg)
acetamiprid	2~10	solvent tea	y = 1871x + 131.2 y = 979.1x + 54.1	0.998 0.992	- −47.7%	2	10
azoxystrobin	1~10	solvent tea	y = 10,187x + 312.2 y = 6146.2x − 732.4	0.997 0.999	- −39.7%	1	/
bifenthrin	5~50	solvent tea	y = 675x − 261.4 y = 840x + 181.2	0.998 0.987	- 28.9%	5	5
carbendazim	0.25~10	solvent tea	y = 4138x + 258.3 y = 6287.8x + 301.2	0.997 0.995	- 52.0%	0.25	5
chlorbenzuron	5~50	solvent tea	y = 5891.4x + 456.4 y = 2174x + 25.6	0.995 0.983	- −63.1%	5	/
chlorpyrifos	2~10	solvent tea	y = 20,466x − 2015.2 y = 14,984x + 1388.6	0.997 0.989	- −26.8%	2	2
cyromazine	0.5~10	solvent tea	y = 29,657x + 55,174 y = 56,556x – 11,775	0.996 0.998	- −86.2%	0.5	/
daminozide	1~10	solvent tea	y = 779.5x + 248.2 y = 1040x + 226.5	0.998 0.983	- 33.4%	1	/
dimethomorph	5~50	solvent tea	y = 17,748.5x + 4056.5 y = 10,408x − 8144.5	0.995 0.996	- −41.4%	5	/
forchlorfenuron	2.5~10	solvent tea	y = 3517.5x − 643.2 y = 2423.1x − 1387.4	0.997 0.999	- −31.1%	2.5	/
metalaxyl	0.1~10	solvent tea	y = 9438x + 4137 y = 16,582x − 1345	0.999 0.999	- 75.7%	0.1	/
methomyl	0.1~10	solvent tea	y = 27,446x − 218 y = 35,271x − 358.5	0.999 0.999	- 28.5%	0.1	0.2
pendimethalin	5~50	solvent tea	y = 1478x + 541.5 y = 2593x + 355.1	0.995 0.985	- 75.4%	5	/
phorat-sulfoxide	3~10	solvent tea	y = 18,744x + 7761 y = 23,661x − 9532	0.999 0.999	- 26.2%	3	/
phosmet	0.5~10	solvent tea	y = 4329x + 276.5 y = 6381.6x − 86.1	0.998 0.995	- 47.4%	0.5	/
phosphamidon	0.2~10	solvent tea	y = 4831x + 633.4 y = 6500x + 418.5	0.997 0.992	- 34.5%	0.2	/
prochloraz	2~10	solvent tea	y = 5231.2x + 457.7 y = 2410.9x − 1573.8	0.999 0.995	- −53.9%	2	/
pyraclostrobin	0.2~10	solvent tea	y = 8792.4x + 7825.4 y = 14,356x + 18,405	0.998 0.991	- 63.3%	0.2	10
triadimefon	0.5~10	solvent tea	y = 28,755x + 4217.6 y = 51,418x − 8735.9	0.997 0.998	- 78.8%	0.5	/
triazophos	0.1~10	solvent tea	y = 112,691x + 4825.7 y = 84,897x + 8800	0.996 0.991	- −24.7%	0.1	/

Note: “/” indicates that the pesticide has no MRLs in tea for China.

**Table 3:** Results of recovery (%, *n* = 5) and relative standard deviations (RSDs).

Pesticide	Add Level (mg/kg)	Average Concentration Test (mg/kg)	RSD (%)	Average Recovery (%)
Acetamiprid	2	1.85	13.1	92.5
	5	4.5	7.5	90.0
	10	11.13	6.3	111.3
Azoxystrobin	1	0.78	4.7	78.0
	5	3.53	5.2	70.6
	10	8.66	3.8	86.6
Bifenthrin	5	3.77	5.7	75.4
	10	8.54	2.9	85.4
	50	44.73	7.1	89.5
Carbendazim	0.25	0.28	7.3	112.0
	2.5	2.04	2.4	81.6
	10	9.76	9.2	97.6
Chlorbenzuron	5	5.58	2.5	111.6
	10	10.65	4.6	106.5
	50	48.67	3.8	97.3
Chlorpyrifos	2	1.57	6.6	78.5
	5	3.85	5.3	77.0
	10	8.04	7.4	80.4
Cyromazine	0.5	0.38	5.5	76.0
	5	4.15	4.7	83.0
	10	7.96	6.4	79.6
Daminozide	1	0.76	7.1	76.0
	5	5.22	3.4	104.4
	10	8.75	4.5	87.5
Dimethomorph	5	5.66	7.3	113.2
	10	9.43	2.9	94.3
	50	52.11	5.8	104.2
Forchlorfenuron	2.5	2.46	4.1	98.4
	5	3.99	8.2	79.8
	10	9.64	4.6	96.4
Metalaxyl	0.1	0.11	6.3	110.0
	2.5	2.89	5.3	115.6
	10	8.77	7.2	87.7
Methomyl	0.1	0.081	5.1	81.0
	5	5.85	4.2	117.0
	10	11.21	9.7	112.1
Pendimethalin	5	5.67	5.6	113.4
	10	9.64	7.8	96.4
	50	47.58	4.8	95.2
Phorat-sulfoxide	3	2.56	8.4	85.3
	5	4.63	6.7	92.6
	10	10.55	1.8	105.5
Phosmet	0.5	0.52	3.9	104.0
	2	1.63	4.1	81.5
	10	8.69	1.7	86.9
Phosphamidon	0.2	0.18	2.3	90.0
	1	0.87	3.6	87.0
	10	9.78	2.7	97.8
Prochloraz	2	1.59	2.5	79.5
	5	3.85	5.1	77.0
	10	10.08	3.2	100.8
Pyraclostrobin	0.2	0.22	5.6	110.0
	5	4.68	4.1	93.6
	10	8.09	4.9	80.9
Triadimefon	0.5	0.45	3.2	90.0
	5	5.38	5.2	107.6
	10	10.44	7.1	104.4
Triazophos	0.1	0.076	3.5	76.0
	5	5.27	4.1	105.4
	10	8.42	8.8	84.2

**Table 4:** The Results of pesticide residue analysis and detection rates in 15 tea samples.

Sample	Detected Pesticides	Screening Concentration (mg/kg)	Full Quantitative Concentration (mg/kg)	Sample Detection Rate (%)
Sample 1	pyraclostrobin	0.197	0.203	66.7
Sample 2	metalaxyl pyraclostrobin	0.144 0.208	0.141 0.311	
Sample 3	ND	ND	ND	
Sample 4	pyraclostrobin	0.434	0.517	
Sample 5	ND	ND	ND	
Sample 6	pyraclostrobin	0.298	0.325	
Sample 7	ND	ND	ND	
Sample 8	pyraclostrobin	0.536	0.642	
Sample 9	pyraclostrobin	0.855	0.919	
Sample 10	ND	ND	ND	
Sample 11	pyraclostrobin	0.375	0.418	
Sample 12	ND	ND	ND	
Sample 13	pyraclostrobin	0.553	0.483	
Sample 14	pyraclostrobin	0.691	0.577	
Sample 15	metalaxyl	0.176	0.180	

## Data Availability

The original contributions presented in the study are included in the article. Further inquiries can be directed to the corresponding author.
